# The Fallacy of the Art of Physic as at Present Taught in the Schools, with the Development of New and Important Principles of Practice

**Published:** 1837-04

**Authors:** 


Art. III.—*The Fallacy of the Art of Physic as at present taught in the Schools, with the Development of new and important Principles of Practice.* By Samuel Dickson, m.d.—*Edinburgh*, 1836. 8vo. pp. 180.

A thinly printed octavo volume, in sixty-six pages of which "the fallacy of the art of physic as at present taught in the schools" was to be demonstrated, we were at first disposed to regard as the offspring of some innocent humorist; but we were disappointed in finding little but ignorance of the science of medicine, as well as of its collateral branches, infinite conceit, and proportionate emptiness. The book is a complete demonstration of the effects arising from a want of knowledge of principles, either of reasoning or medicine. "Fever, remittent or intermittent, comprehends every shape or shade which disorder can assume." (P. 2.) This is the new doctrine, of the truth of which Dr. Dickson is thoroughly convinced, and the triumph of which he foresees. "Disease," says our author, "is simply increase or diminution of the proper functions of health." The admission of the correctness of this definition would be an important step gained by Dr. Dickson, in the establishment of his new doctrine. As it is a mere assertion of his opinion, we meet it simply with a contrary assertion. If, when its author has attempted to prove its universality by including within it cases of *perverted* nutrition; if, when he has paid due attention to the large class of what are termed organic diseases, with which the internal evidence of his book shows him to be unacquainted, he still retains the same opinion, we may then consider his definition worthy of consideration.

Without any attempt to prove that the distinction of fevers into symptomatic and idiopathic is not grounded in truth, and without any allusion to the phenomena by which these are supposed to be distinguished, Dr. Dickson says, that "structural lesion, so frequently but erroneously associated with disease as a cause, is not even necessary to the production of disorder." (P. 6.) Thus dogmatically is the question decided. We read of the pride of knowledge, but we have rarely seen so well demonstrated on how small a foundation of the latter the former may be maintained.

Dr. Dickson proceeds next to the "causes of disease." He tells us that "they are *infinite;* they affect the body principally from without, *acting upon it, in the first place, through the different modifications of nervous perception,* and seldom, *if ever,* originating in any one organ of the body; for, before a vital part can be materially implicated, all must be more or less involved. Such, however, is not the doctrine of the schools." (P. 6.) In this conclusion we cordially agree, being quite ignorant of the school in which are taught the different modifications of nervous perception through which the causes of disease affect the body. The mere assertion which constitutes the remainder of the foregoing sentence, and which is involved in the question to which we have previously alluded, we treat as we should the assertion that Dr. Dickson had acquired any knowledge in "the schools:" we regard it as valueless, in default of any evidence.

The next step in Dr. Dickson's inductive process is the "nature of disease." The following sentence is the basis of the new doctrine: "The phenomena of organized existence, like the chemical combinations of unorganized matter, *can only take place by means of change of temperature.* By moderate alternations of this, every action and appetite of animal life is regulated in health. Through its excessive increase or diminution alone can disease be produced: in either case, it is alternate or remittent." This is the proposed substitute for the theory of medicine as at present taught in the schools.

Probably, the simplest method of explaining to our author the fallacy of his proposition will be to state it, consistently with fact, and to append to it his own deduction. Chemical action is influenced by change of temperature; vital actions are also influenced by such changes; but both the chemical and vital actions give rise to changes of temperature; therefore (such is Dr. Dickson's *sequitur,*) the phenomena of organized existence, like the chemical combinations of unorganized matter, can *only* take place *by means of* change of temperature. Without admitting the identity of chemical and vital action, we give our author the full benefit of the admission. A simple illustration may render him more conscious of his mistake. Sulphuric acid and water, when mixed together at the ordinary temperature, give rise to the development of previously latent caloric; brisk exercise causes warmth of the body. Heat the water, or let the individual exercise himself in a very elevated temperature, and the chemical and vital actions are attended with a greater development of heat: therefore, Dr. D. would say, chemical and vital actions can only take place by means of change of temperature. But the application of this discovery, both to health and disease, is of the utmost importance; and, first, to health. "By moderate alternations of this, (i. e. temperature,) every action and appetite of animal life is regulated;" e. g. the desire and the act of writing his most curious work are solely ascribable to change of temperature. The application of the principle to disease is above stated: "Through its excessive increase or diminution alone can disease be produced." This sentence contains its own refutation. Where is the *excessive* increase or diminution of temperature in the majority of diseases? But we will give our author the word, as well as the advantage derivable from his own illustrations; remembering that his argument is as follows:—All diseases are caused by alternate or remittent variations of temperature; therefore, all diseases are remittent or intermittent fevers. Had he spoken of these variations as the mere accompaniments of some diseases, we should not very much have differed from him. As his doctrine is applicable to *all* diseases without exception, he will allow us to select from his illustrations those which we think the most appropriate. He asks, triumphantly anticipating an affirmative reply, "Is the gout a remittent fever simply?" (p. 19;) and then quotes Sydenham's description of it, where pain is said to be the first symptom, and not a word is said of change of temperature until the severity of "the first attack has continued twenty-four hours," when the patient "begins to perspire." In his anxiety to prove the remissions in gout, which we willingly accord, he has unfortunately overlooked the fever. The next illustration we quote entirè, as a favorable specimen of the author's style, and of the general character of his evidence.

"Is the stone remittent? Assuredly there are times of the day when the subject of it is better and worse, and this without reference to the period of micturition. Public rewards have from time to time been given for secret remedies in this disease. These remedies by their influence over fever, *without which stone cannot be produced,* have simply given the bladder the power of retaining the stone with less uneasiness. I have shewn that rheumatism and gout are remittent diseases. Calcareous deposition takes place in the course of these, and writers have accordingly marked their analogy to stone. Calculary depositions may take place in any structure of the body. Salivary calculi are common; pulmonary calculi I have seen. The liver, gall bladder, and kidneys are often the seat of stone. Occurring in the course of an artery, we term it ossification. The false cartilages found in joints are a mere variation, and the subjects of *all* are the subjects of *remittent fever.*" (P. 20, 21.)

Here, dogmatism and ignorance seem to be striving for the preeminence, and we know not to which to accord it.

We need not pay any particular attention to the "mode of cure," which forms the next department of Dr. Dickson's work. Those, however, who are satisfied with his theoretical views, will be gratified to hear that he "knows not a form of disease in which he has not found quinine useful; not one where not only has it failed to accomplish his intentions, but where it has actually aggravated the existing symptoms. From mercury, iron, silver, arsenic, and a host of other remedies, he has obtained similar results." (P. 25.) By quinine he "has often succeeded in the cure of consumption," (41.) But prussic acid and nitrate of silver are, in our author's hands, like the "universal medicine." The diseases in which he has successfully employed them, we will quote in Dr. D.'s own words:

"When discreetly administered, I know no two remedies so universally applicable to the treatment of diseases. With one or other I have successfully encountered tremor of every species, comprehending stutter, stagger, cardial palpitation and remission. I know no remedy equal to them in spasm of whatever name or nature, from asthma and urethral stricture to epilepsy. I have experienced their value in every variety of morbid sensation, from mental exaltation and depression to mania, and I have with both completely cured every kind of palsy, from simple strabismus to loss of speech and locomotion. In hæmorrhages, catarrhs, and other discharges, prussic acid is unrivalled by any other article of the materia medica. Cough, pulmonary congestion, and every kind of varicose fulness, with pectoral, gastric, lateral, and abdominal pains, will generally yield to one or the other. Consumption I have cured by both. From every kind of fainting-fit, and every change of structure and secretion, including flatulence, they are useful beyond any other remedy with which I am acquainted. To sum up all, they can cause and cure *fever.*" (P. 42.)

The only scholastic source with which we are acquainted, whence the foregoing paragraph can have derived its peculiar character, is, certain advertisements of certain pseudo-medical adventurers, which regularly appear in weekly newspapers. Need we a stronger proof of the value of pathology than the foregoing folly? of the utility of "that bauble of Laennec, the stethoscope," (p. 16,) than the laconically stated cures of consumption? or of the advantages of common sense, than the want of it which is here manifested?

The remainder of Dr. Dickson's book is devoted to the consideration of several diseases, all of course illustrative of the previous theory. Our reasons for not noticing them are that the theory is a flimsy illusion; that what is already known it is needless to repeat; and that our confidence in Dr. Dickson's judgment is so small, that it would be worse than useless to notice the rest.

The claim to novelty which our author puts forth in the early part of his volume, it would be injustice not to accord. The work is one quite *sui generis*, written by an individual to whom the art of reasoning is an impenetrable secret; to prove the incorrectness of a science with the principles and details of which he is evidently unacquainted. A book of this kind requires the most unqualified condemnation: we should otherwise have left it to its merited oblivion.

